# Prevalence of Epidermal Growth Factor Receptor Mutations in Patients with Non-Small Cell Lung Cancer in Turkish Population

**DOI:** 10.4274/balkanmedj.2017.0297

**Published:** 2017-12-01

**Authors:** Gaye Güler Tezel, Ebru Şener, Çisel Aydın, Sevgen Önder

**Affiliations:** 1 Department of Pathology, Hacettepe University School of Medicine, Ankara, Turkey; 2 Clinic of Pathology, Erzurum Regional Training and Research Hospital, Erzurum, Turkey

**Keywords:** Non-small cell lung cancer, epidermal growth factor receptor mutation, ethnicity

## Abstract

**Aims:**

*Epidermal growth factor receptor* mutation analysis in non-small cell lung cancer is important for selecting patients who will receive treatment with tyrosine kinase inhibitors. In this study, we aimed to investigate the prevalence of epidermal growth factor receptor mutations and mutation patterns in the Turkish population.

**Methods:**

We retrospectively reviewed molecular pathology reports of 959 cases with lung cancer analysed for *epidermal growth factor receptor* mutations. We analysed all four *epidermal growth factor receptor* *exon* mutations using a real-time polymerase chain reaction platform.

**Results:**

In this study, the *epidermal growth factor receptor* mutation rate in the Turkish population was 16.7% (160 of 959). The *epidermal growth factor receptor* mutation frequency was significantly higher in women (37.1%, n=96) than in men (9.1%, n=64) (p<0.001). In addition, the *epidermal growth factor receptor* mutation rate was higher in the adenocarcinoma histologic type (p<0.001). Patients with mutations were older than those without mutations (p=0.003). The most frequent mutations were *exon* 19 deletions (48.8%, 78/160) and *exon* 21 L858R point mutations (38.1.1%, 61/160). We also detected compound mutation patterns in three cases (1.9%).

**Conclusion:**

The prevalence of *epidermal growth factor* receptor mutations in the Turkish population was slightly higher than that in the Caucasian population and lower than that in the East Asian population. The results of this study may provide guidance in personalized therapy of non-small cell lung cancer in the Turkish population.

Lung cancer is one of the most common causes of cancer deaths in men and women (1). It is the most common cancer in men and the fifth most common cancer in women in Turkey (2). Most lung cancer patients receive their diagnosis when their cancer is already advanced or metastatic, and the 1-year survival rate is unfortunately less than 15% if the cancer is not treated (3).

Epidermal growth factor receptor (*EGFR*) is a receptor tyrosine kinase (TK) of the ErbB family. Mutations in the TK domain of *EGFR* lead to autophosphorylation and therefore a continuous activation in the TK region. Consequentially, abnormal expression of *EGFR* results in tumour cell proliferation, angiogenesis, invasion, metastasis and inhibition of apoptosis (4,5).

In the TK domain of *EGFR*, activating somatic mutations from *exon*s 18 to 21 were first found in patients with lung adenocarcinoma in 2004 (6). In the 2004 studies, most of the lung cancer patients responding to *EGFR* TK inhibitors (TKIs), such as erlotinib and gefitinib, reportedly had *EGFR* mutations (6,7). Some clinical characteristics (Asian origin, never smoked, female gender and histologic adenocarcinoma subtype) are associated with the presence of *EGFR* mutations in patients with non-small cell lung cancer (NSCLC) (8). However, selection of the patients to be treated should be according to the *EGFR* mutation analysis results, rather than these clinicopathological characteristics (9).

In earlier studies, the *EGFR* mutation frequency reportedly varied proportionally among different ethnic groups (10,11). To the best of our knowledge, no study has investigated the prevalence of *EGFR* mutations and mutation profiles in a large series of the Turkish population. The purpose of this study was to identify *EGFR* mutation prevalence, mutation types and clinicopathological characteristics of these patients in the Turkish population.

## MATERIALS AND METHODS

### Patients and samples

Ethics committee approval was received for this study from the ethics committee of Erzurum Regional Training and Research Hospital (Date of approval: 21 June 2016; number: 37732058-53-4099). In this study, we retrospectively reviewed molecular pathology reports from 963 cases with NSCLC analysed for *EGFR* mutations at the Department of Pathology, Hacettepe University, from December 2011 through February 2015. However, in four cases, we did not conduct *EGFR* mutation analysis, because we could not retrieve sufficient and/or good quality DNA. Therefore, 959 patients were included in the study. The median age of the patients was 60 (range 22-87); 250 patients (26.1%) who were tested for *EGFR* mutations were diagnosed in our pathology department and 709 patients (73.9%) were diagnosed in other pathology laboratories from different regions of Turkey (Samsun, Erzurum, Trabzon, Gaziantep, etc.) and referred to our laboratory for mutation analysis. Specimens diagnosed as adenocarcinoma (698 cases, 72.8%), NSCLC not otherwise specified (NSCLC-NOS) (243 cases, 25.3%) and squamous cell carcinoma (18 cases, 1.9%) were included.

We obtained tumour samples for *EGFR* analysis from different origins, including primary lung lesions or metastatic lesions. We used formalin-fixed paraffin-embedded (FFPE) tissues, cell blocks and stained cytology slides for *EGFR* mutation testing.

### DNA extraction and quantification

The pathologist marked the tumour samples on the haematoxylin and eosin-stained sections to choose the tumour-rich areas, and then these areas were manually macrodissected on 8-mm-thick unstained sections to eliminate as many non-malignant, stromal and contaminating inflammatory cells as possible. We used single-use sterilized scalpels to prevent contamination. Genomic DNA was isolated from FFPE and cell blocks using a QIAamp DNA FFPE tissue kit (Qiagen, Germany) according to the manufacturer’s instructions. For stained (either haematoxylin and eosin, Papanicolaou or Giemsa) cytology slides, DNA was extracted using the phenol-chloroform method (12). Samples that contained at least 25% tumour cells were tested. The genomic DNA concentration was quantified using spectrophotometry (NanoDrop 2000, Thermo Scientific, Waltham, MA).

### Real-time polymerase chain reaction

We analysed all four *EGFR* *exon* mutations (*exon*s 18, 19, 20 and 21) using EntroGen’s *EGFR* mutation analysis kit on an Applied Biosystems StepOnePlus real-time polymerase chain reaction (PCR) platform. Mutational analysis was accomplished for all the samples as described in the kit procedure.

### Statistical analysis

The data were analysed using SPSS version 18 for Windows. We expressed continuous variables as median and categorical data as percentages. We used the chi-square test to compare *EGFR* mutation status with clinicopathological characteristics. Differences in continuous measurements between two groups (*EGFR* mutation status and age) were examined by the Student’s t-test. We considered a two-tailed p<0.05 to indicate statistical significance.

## RESULTS

Of the 959 samples (700 men, 259 women), 698 were adenocarcinoma, 243 were NSCLC-NOS and 18 were squamous cell carcinoma. The overall mutation rate was 16.7% (160 of 959). *EGFR* mutations were significantly more frequent in females (37.1%) (n=96) than in males (9.1%) (n=64) (p<0.001). We found that patients with *EGFR* mutations were significantly older than those without *EGFR* mutations (p<0.001). The distribution of mutation cases according to diagnosis were 142/698 (20.3%) adenocarcinoma, 18/243 (7.4%) NSCLC-NOS and 0/18 (0%) squamous cell carcinoma. We observed a statistically significantly higher *EGFR* mutation prevalence in adenocarcinomas (p<0.001). Mutations were detected in 117/677 (17.3%) primary and 43/282 (15.2%) metastatic pulmonary tumour samples. We did not see any differences between primary and metastatic samples (p=0.441). In addition, no significant difference was found between FFPE (resections materials and biopsies) tissues and cytologic materials in terms of the rate of *EGFR* mutation (p=0.927).

Unfortunately, of 959 patients, we could determine only 35 patients’ smoking histories. Although the difference was not statistically significant, *EGFR* mutations were more frequent in non-smoking patients (10/25) than in smokers (2/10). Clinical characteristics of 959 patients with NSCLC who were subjected to *EGFR* mutation analysis and their association with *EGFR* mutations are summarized in Table 1.

Of 160 mutation cases, the most common mutation was an in-frame deletion in *exon* 19, comprising 48.8% (78/160) of all mutations found, followed by a point mutation (L858R) in *exon* 21, comprising 36.9% (59/162) of mutations. These two most common drug-sensitive mutations comprised 85.7% of all mutation cases. The other rarely seen drug-sensitive mutations were *exon* 18 G719X, observed in nine cases (5.6%), and an *exon* 21 L861Q mutation, seen in two patients (1.2%). In addition, an *exon* 20 mutation was found in nine cases (5.6%); eight were patients with *exon* 20 insertion mutations, and one case had an *exon* 20 T790M point mutation. Instead of classical mutation patterns, we detected compound mutation patterns in three cases (1.9%). In two of these cases, the *exon* 19 deletion and *exon* 20 T790M point mutation were detected together, while an *exon* 21 L858R point mutation and *exon* 18 G718X point mutation was detected together in one patient. *EGFR* mutation status patterns are shown in Table 2.

## DISCUSSION

Determining the presence of *EGFR* mutations is crucial in terms of selecting patients with advanced or metastatic lung cancer who will receive treatments with TKIs, such as gefitinib or erlotinib, because activating mutations in the *EGFR* gene in lung tumours is associated with an effective and dramatic response to TKIs (6). In this study, we tried to determine the *EGFR* mutation frequency of 959 patients with NSCLC and found it to be 16.7% in the Turkish population. We found a higher *EGFR* mutation frequency in women, the adenocarcinoma histological subtype and the elderly population.

This is the first comprehensive study investigating the *EGFR* mutation rate in the Turkish population. Because our study provides an *EGFR* mutation profile in a wider patient population, and tumour samples used in the *EGFR* mutation analysis were brought from hospitals in different regions of Turkey, we believe that our results reflect the *EGFR* mutation rate in the Turkish population more accurately.

In the earlier studies, the *EGFR* mutation prevalence reportedly varied between ethnic groups. For example, this rate was found to be 10%-15% in Caucasian patients (10), whereas it was reported as 40%-60% in East Asian patients (11). The mutation rate we found in Turkish patients (16.7%) is slightly higher than that reported in earlier studies on Caucasians. Similar to our results, in a study conducted in 2009, the *EGFR* mutation rate of Caucasians (Spanish patients) was reported as 16.6% (13).

The frequency of *EGFR* mutation depends not only on ethnicity but also on gender, NSCLC histological type and smoking status. In many studies, *EGFR* mutation rates in women have been reported to be higher than those in men (8). In parallel with these results in the literature, we found the frequency of *EGFR* mutation to be higher in female than in male patients (37.1% vs. 9.1%).

We found the rate of *EGFR* mutation to be 20.3% in adenocarcinomas and 7.4% in NSCLC-NOS. In line with earlier studies, we found the mutation rate in the histological subtype of adenocarcinoma to be higher (p<0.001) (8). However, we did not find *EGFR* mutations in any of the 18 cases diagnosed with squamous cell carcinoma. In a few studies, the rate of *EGFR* mutation reported was very low in squamous cell carcinoma (14). However, consistent with our results, other studies could not detect any *EGFR* mutations in squamous cell carcinoma (10).

The most common mutations found in our study were an in-frame deletion (n=78, 48.8%) in *exon* 19 and a point mutation (L858R) in *exon* 21 (n=61, 38.1%). These two mutations are sensitive to treatment and comprise 89.9% of all mutation cases; this high rate is consistent with the literature (6,7). Exon 18 G719X, *exon* 21 L861Q, *exon* 20 mutations and compound mutations that we have detected in three patients are rarely seen mutation types. Although *exon* 20 mutations are usually associated with resistance to *EGFR* TKIs, recent studies show that new covalent inhibitors have shown efficacy against relapsing disease during previous treatment with an existing *EGFR* inhibitor (15,16). Other studies have determined the rate of compound mutation to be 7% (17). Limited data are available in the literature about the response of these mutations to treatment.

The *EGFR* mutation rate is reported to be higher in non-smokers than in smokers (6,7). We identified the smoking status of only 35 of 959 patients. The most important reason of our inability to detect a correlation between the smoking status and the frequency of *EGFR* mutation is that we could only access the smoking history of very few patients.

Aging causes an accumulation of genetic alterations by reducing stem cell fitness; additionally, the prevalence of oncogenic mutations increases with age (18). We found that patients with *EGFR* mutations were older than those without mutations. In parallel with our results, there are studies in the literature suggesting that the rate of *EGFR* mutation is higher in the elderly population (19).

Discordances between primary tumours and corresponding metastatic tumours in terms of *EGFR* mutation status are extremely rare (9). According to the *EGFR* mutation results of our study, there was no significant difference between primary and metastatic samples. In this study, we observed no significant difference in the mutation rates of cytological specimens and FFPE tissues (resections and biopsies). This may have been due to the sensitive technique (real-time PCR) that we used as the mutation analysis method. Similar results were obtained in studies that also used sensitive techniques (20).

As a result, we found the *EGFR* mutation rate in the Turkish population to be 16.7%. In line with earlier studies in the literature, we found a higher mutation rate in the adenocarcinoma histological subtype, comparing to all other subtypes. The results of this study may provide guidance in determining a personalized treatment regimen in NSCLC by giving us genetic information about lung cancer in the Turkish population.

## Figures and Tables

**Table 1:**
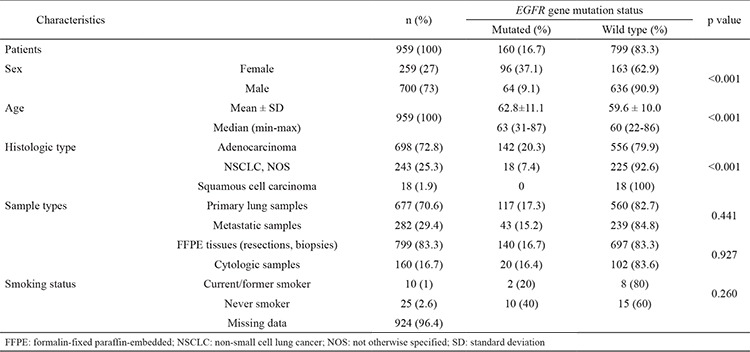
Characteristics of all patients according to *EGFR* gene mutation

**Table 2:**
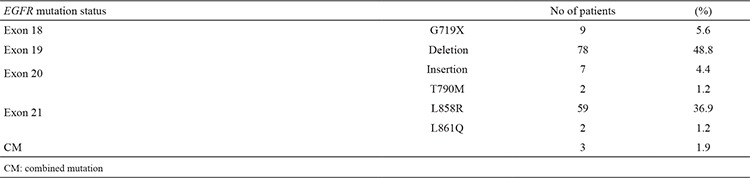
Type of *EGFR* gene mutation and their distribution in the study population
